# Plasma prolactin and breast cancer risk: a meta- analysis

**DOI:** 10.1038/srep25998

**Published:** 2016-05-17

**Authors:** Minghao Wang, Xiujuan Wu, Fan Chai, Yi Zhang, Jun Jiang

**Affiliations:** 1 Breast Disease Center, Southwest Hospital, Third Military Medical University, Chongqing, 400038, China

## Abstract

Breast cancer is the most common cancer among women, and its incidence is on a constant rise. Previous studies suggest that higher levels of plasma prolactin are associated with escalated risk of breast cancer, however, these results are contradictory and inconclusive. PubMed and Medline were used to search and identify published observational studies that assessed the relationship between plasma prolactin levels and the risk of breast cancer. The pooled relative risks (RRs) with 95% confidence intervals (CIs) were calculated using a fixed-effects or random-effects model. A total of 7 studies were included in our analysis. For the highest versus lowest levels of plasma prolactin, the pooled RR (95% CI) of breast cancer were 1.16 (1.04, 1.29). In subgroup analyses, we found a positive association between plasma prolactin levels and the risk of breast cancer among the patients who were postmenopausal, ER^+^/PR^+^ or *in situ* and invasive carcinoma. However, this positive association was not detected in the premenopausal and ER^-^/PR^-^ patients. In conclusion, the present study provides evidence supporting a significantly positive association between plasma prolactin levels and the risk of breast cancer.

As a notorious malignant disease, breast cancer represents the most common type of cancer among women. Unfortunately, the incidence rate of breast cancer has been rising rapidly over the past decades both locally and globally. For instance, breast cancer is currently the second leading cause of cancer-related death in the United States^1^. Despite many promising advances over the past several years, our understandings of the initiation, maintenance, and metastasis of breast cancer remain far from thorough. Many previous studies primarily focused on the etiology, oncogenesis and treatment of breast cancer. However, it is believed that hormones in women may also play an important role in initiating and promoting breast cancer^2^. Particularly, prolactin is such a well-defined lactogenic hormone, which promotes the proliferation of breast epithelial cells and the differentiation of alveoli^3^. Several breast cancer risk factors, such as nulli-parity and high mammographic breast density, have been identified to be correlated with increased levels of serum prolactin^4^,^5^. Intriguingly, multiple pieces of experimental data reveal that prolactin can induce cell proliferation, tumor vascularization and cell motility, which function independently and synergistically to promote late-stage carcinogenesis of breast cancer and to enhance its propensity to metastasize to distant organs^6^,^7^.

Notably, Tworoger *et al*. showed evidence from elegant studies indicating that higher levels of plasma prolactin were associated with an increased risk of breast cancer in both premenopausal and postmenopausal women, which was especially prominent for the estrogen receptor+/progesterone receptor+ cancer type^8^,^9^. Collectively, these results strongly suggest that plasma prolactin functions as an important pathogenic factor that contributes to the etiology of breast cancer. However, another study published in 1992 demonstrated that there was no significant relationship between the risk of breast cancer and plasma prolactin levels in either pre- or postmenopausal women^10^. Consequently, although the association between plasma prolactin levels and risk of breast cancer has been extensively interrogated by many independent studies, it remains to be definitively determined. In order to clarify these conflicting results, we performed a meta-analysis based upon previously published literature to comprehensively examine the relationship between plasma prolactin levels and the risk of breast cancer.

## Methods

### Literature Search

PubMed and Medline databases were used to identify relevant studies that were published no later than February 2016 with focused on the association between plasma prolactin levels and the risk of breast cancer. The following phrases and terms were used in the searching strategy: “serum prolactin” or “plasma prolactin” in combination with “breast cancer” or “breast carcinoma”. References of the relevant reviews were also scrutinized.

### Study selection

Studies were deemed eligible if they met the following inclusion criteria: 1, designed as a case-control or cohort study; 2, assessed the association between serum/ plasma prolactin levels and the risk of breast cancer; 3, all cases of breast cancer diagnosed and verified by pathological biopsies or other standard methods, with controls being females without breast cancer; and 4, reported relative risk (RR) or odds ratio (OR) and the corresponding 95% CI for the highest versus the lowest levels of plasma prolactin.

According to the above-described inclusion criteria, the titles and abstracts were reviewed to determine whether a candidate study was potentially eligible. For those that were difficult to determine, a full-text assessment was conducted. The literature selection was conducted in replicates by two independent authors. All disputes, if any, were resolved by discussion.

### Data extraction and quality assessment

The following information was collected from the included studies: the last name of the first author, publication year, sample size and RR from multivariable models for the highest versus the lowest category of plasma prolactin levels with the corresponding 95% CI.

Evaluation of the study quality was conducted using the Newcastle-Ottawa quality assessment scale (NOS)^11^. On a score scale from 0 to 9, a study with 7 or more stars was considered as high-quality.

### Statistical analysis

STATA version 12.0 (Stata Corporation, College Station, Texas, USA) was used to analyze the data. The combined RR and the corresponding 95% CI were used to measure the association between plasma prolactin levels and the risk of breast cancer using a fixed-effects model or random-effects model. Homogeneity test was performed with Q statistic and I^2^ statistic. Subgroup analysis was performed to identify various factors that influenced the pooled estimate. Additionally, sensitivity analysis was used to investigate the influence of a single study on the overall risk estimate by excluding one study at a time. Publication bias was detected by Begg’s and Egger’s test. P-value less than 0.05 was considered as statistically significant.

## Results

### Literature search and baseline characteristics

**Figure 1:**
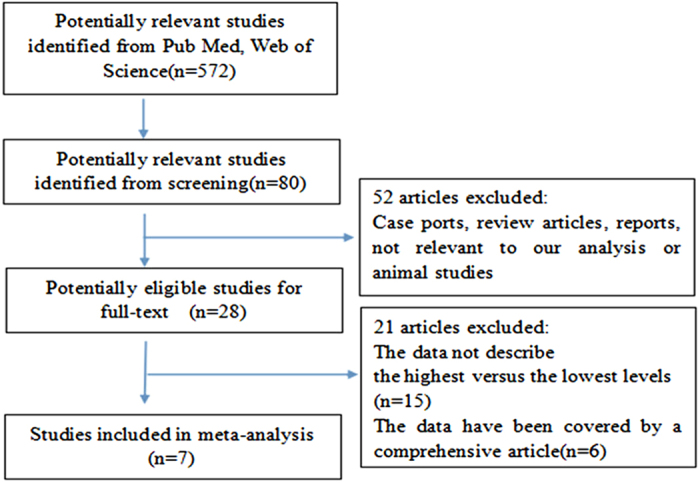
Flow chart of study selection.

We initially identified 572 potentially eligible articles from PubMed and Medline. However, most of them were excluded primarily because they were review articles, reports, animal studies or irrelevant to our analysis. After reviewing the remaining 28 articles, we decided to exclude 15 studies that did not report the highest versus the lowest levels of plasma prolactin and 6 studies that reported duplicate data from the same participants. Ultimately, we selected 7 eligible studies (1 case-control and 6 cohort studies) that involved 12275 participants and 6388 cases^10^,^12^,^13^,^14^,^15^,^16^,^17^. Figure 1 presents the flow chart of study selection.

The baseline characteristics of the included 7 studies were presented in Table 1. These studies were published between 1992 and 2015 in English. The RRs in each study were estimated based on the highest versus the lowest plasma prolactin levels. Intriguingly, the results from one study indicated that elevated levels of plasma prolactin were associated with an increased risk of breast cancer *in situ*^17^, whereas the other 6 studies reported no such correlation^10^,^12^,^13^,^14^,^15^,^16^. RRs of all the studies were adjusted for a wide range of potential confounding factors, including age, BMI, age at menarche and menopause, family history of breast cancer, duration of postmenopausal and hormone use. It has to be noted that all of the included studies in our analysis were considered as high quality according to our criteria.

### Main analysis

**Figure 2:**
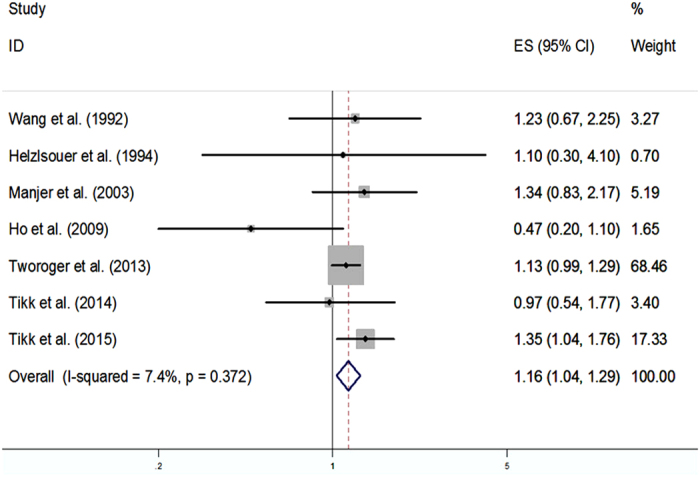
Meta-analysis of 7 studies that assess the association of plasma prolactin levels with breast cancer risk.

The adjusted RRs for each study, as well as for all the studies combined, were described in relation to breast cancer risk with the highest versus the lowest levels of plasma prolactin in Fig. 2. Among these 7 selected studies, 1 suggested a positive association between plasma prolactin levels and risk of breast cancer^17^. However, the remaining studies revealed no such association^10^,^12^,^13^,^14^,^15^,^16^. When combined using a fixed-effects model, we revealed a statistically significant positive relationship between plasma prolactin levels and risk of breast cancer, with the RR (95% CI) being 1.16 (1.04, 1.29) (P < 0.05). Notably, there was no evidence of heterogeneity (P = 0.372, I^2^ = 7.4%).

### Subgroup Analysis

Table 2 shows the results of a subgroup analysis stratified by menopause status, ER/PR status, morphology and study design. Intriguingly, the RR (95% CI) was determined to be 1.27 (1.13, 1.43) and 0.99 (0.84, 1.16) for postmenopause and premenopause patients, respectively, thereby revealing a positive association between plasma prolactin levels and risk of breast cancer among the postmenopause patients, but not in the premenopause women. In addition, our results indicated a positive correlation between plasma prolactin levels and risk of breast cancer among the ER+/PR+ patients (RR (95% CI) as 1.26 (1.04, 1.53)), although no association was uncovered in the ER-/PR- patients (RR (95% CI) as 1.0 (0.70, 1.43)). Importantly, the RR (95% CI) was calculated as 1.32 (1.07, 1.63) and 1.29 (1.04, 1.61) for invasive carcinoma and *in situ* carcinoma, respectively. Lastly, our results also revealed a positive correlation between plasma prolactin levels and risk of breast cancer among the cohort studies (RR (95% CI) as 1.17 (1.05, 1.31)).

### Sensitivity Analysis

**Figure 3:**
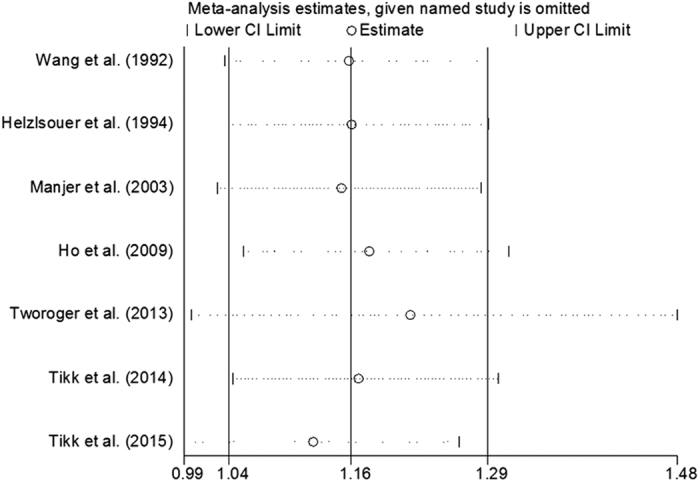
Forest plot for sensitivity analysis.

Our results showed that the combined RRs were essentially unaltered as no single study included in our analysis significantly compromised the results except for the study conducted by Tworoger *et al*. in 2013 and by Tikk *et al*. in 2015. However, after removing these two studies, the positive relationship between plasma prolactin levels and risk of breast cancer was still identified, which indicated statistical stability and reliability of our results (Fig. 3).

### Publication Bias

**Figure 4:**
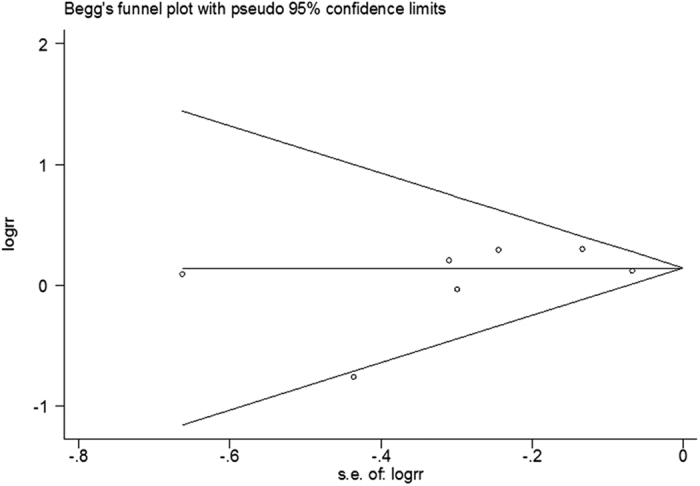
Forest plot for publication bias.

The funnel plot showed a low probability of publication bias (Fig. 4). Consistently, the Egger’s regression test indicated little evidence of publication bias (P = 0.576).

## Discussion

In the present study, 7 eligible studies were identified and combined to interrogate the association between plasma prolactin levels and the risk of breast cancer. Our results suggested that plasma prolactin levels were associated with an increased risk of breast cancer, particularly for those who are diagnosed as the ER+ subtype and postmenopausal patients.

Heterogeneity often occurs as a result of various different factors, thereby representing a deteriorating challenge for meta-analysis^18^. Such heterogeneity often results from different study designs and assays of plasma prolactin. However, our results revealed little evidence of heterogeneity among the included studies. Although our eligible studies reported results from distinct ethnic areas, they all focused on the Western populations, which considerably resembled each other in terms of routine lifestyle and incidence of breast cancer. Importantly, all eligible studies were published in English and exhibited high scores after quality assessment. The risk estimate for each individual eligible study was adjusted to account for a variety of potential confounding factors, such as age, BMI, age at menarche and menopause, family history of breast cancer, duration of postmenopausal hormone use.

We conducted a sensitivity analysis to evaluate the possible impact that each included study may impose on the pooled RR, and identified that the studies published by Tworoger *et al*. in 2013 and by Tikk *et al*. in 2015, when removed from our analysis, can greatly change the pooled RR. As shown in Fig. 3, the weight of these two particular studies were much higher than that of others, although the underlying mechanism leading to this phenomenon remains largely unclear. Possible explanations may lie in the fact that these two studies had the largest sample size, longest duration of follow-up and adjustment for many potential confounding factors. Despite the inclusion of these studies, however, the positive association between plasma prolactin levels and the risk of breast cancer remains to be identified, which suggests that our results are robust.

We also performed subgroup analyses to assess how various factors influence the relationship between plasma prolactin levels and risk of breast cancer. Our results demonstrated that the positive association was not changed by cohort study design or the morphology of breast cancer. In contrast, however, such a positive association was not identified among the premenopause women (RR (95% CI) as 0.99 (0.84, 1.16)). This may be possibly explained by the great number of postmenopausal women who participate in the blood draw in our studies. New cases that were added to our pool were more likely to be postmenopausal at diagnosis. Combined together, these results indicated that measurement of plasma prolactin levels during the postmenopausal period may be more important and accurate in predicting the risk of breast cancer than during the premenopausal period.

In addition, our analysis revealed a positive association between plasma prolactin levels and the risk of breast cancer in patients who were positive for ER+/PR+, but not for the other ER/PR status. This is not a surprising observation because a previous study has demonstrated that prolactin is highly likely linked to the development of ER+ tumors and, importantly, estradiol and prolactin exert a synergistic effect on cell proliferation^19^. Furthermore, despite the paucity of ER+ tumors in transgenic mouse models, increased expression levels of active prolactin may potently induce the development of ER+ tumors^20^. Indeed, these results are consistent with two previous *in vitro* studies demonstrating that long-term prolactin exposure elicits elevated ER expression^19^,^21^.

Meanwhile, our systemic interrogation uncovered a positive association between plasma prolactin levels and the risk of breast cancer in both primary and invasive tumors. Previous literature has demonstrated that prolactin plays an indispensable role in the initiation and development of breast cancer through inducing cell proliferation and inhibiting apoptosis^22^. Moreover, prolactin also functions to enhance angiogenesis and cell migration, which may contribute considerably to cancer metastases^23^. Indeed, escalated plasma prolactin levels are often found in patients with developed and late-stage cancers. However, there were only two studies included in each subgroups, respectively.

It has to be noted that although we identified a significant positive association between plasma prolactin levels and the risk of breast cancer, the mechanisms underlying this relationship remain entirely elusive. Prolactin plays an essential role in the growth of mammary glands and the process of lactation. Notably, a previous study has shown that exogenous administration of prolactin strongly increases the likelihood of mammary tumor formation, and, consistently, suppression of prolactin levels exerts an opposite effect in animal models^24^. It is additionally demonstrated that prolactin facilitates the growth of both normal and malignant breast cells *in vitro*^25^. In line with this, results from multiple lines of studies showed that expression levels of prolactin and prolactin receptors from breast cancer cells and tissues were much higher than normal tissues^21^,^26^. Additional work also reported that, other than the robust tumor-promoting function, prolactin can serve to augment the number of S phase-cells, boost cell proliferation frequency and increase levels of cyclin D1 in breast cancer cell lines^19^.

Our results need to be interpreted with precautions for several reasons. Foremost, circulating prolactin in human plasma exists in multiple distinct forms, which may assume different biological functions^27^. Indeed, no previous studies have reported how various forms of prolactin differentially influence the risk of breast cancer development. Second, as a strong circadian rhythm hormone, the production level of prolactin is always elevated after a noontime meal^28^. Due to the paucity of relevant publications, we were not able to assess whether the rhythm of prolactin levels is correlated with the risk of breast cancer in the present study. In addition, only one case-control study is included, which inevitably imparts a recall and selection bias. Furthermore, despite optimal adjustments, residual confounding factors from the original studies could not be controlled. All included studies were based on the Western population, and it thus remains to be fully determined whether our findings are supported by other distinct populations.

Despite the above-mentioned drawbacks of the analysis, the present study has some important merits that deserve special attention. Foremost, our study represents the first attempt to assess the relationship between plasma prolactin levels and the risk of breast cancer by combining all observational studies. It is widely accepted that analysis of individual studies harbors insufficient statistical power. In contrast, we identified and combined all eligible studies in the present study that together constitutes a large sample size, which provided more reliable results with greatly enhanced statistical power. With this strategy, we revealed a positive association between plasma prolactin levels and the risk of breast cancer. Our observations further supported that, in addition to its utility as a biomarker, prolactin may also be a risk factor for breast cancer.

## Conclusions

Our results demonstrated a positive relationship between plasma prolactin levels and the risk of breast cancer. Particularly, such correlation is markedly prominent for those who are diagnosed as the ER+/PR+ subtype and postmenopausal patients.

## Additional Information

**How to cite this article**: Wang, M. *et al*. Plasma prolactin and breast cancer risk: a meta- analysis. *Sci. Rep.* **6**, 25998; doi: 10.1038/srep25998 (2016).

## Figures and Tables

**Table 1:** The characteristics of included studies on the relationship between plasma prolaction levels and breast cancer risk.

Study (Year)	Country	Study period	Study design	Age range	Sample Size(n) case/control or participants	Prolaction levels ange(mg/ml)	Adjusted RR (95% CI) (highest vs. lowest)	Variables used in multivariate model
Wang *et al*.^10^	England	1968–1976	Cohort	18–90	111/3776	Pre I (6.9–9.5) II (9.5–12.5) III (9.5–12.5) IV (>16.9) Post I (4.3–6.0) II (6.0–7.5) III (7.5–9.9) IV (>9.9)	Pre 1.07 [0.51, 2.23] Post 1.63 [0.57, 4.71]	Age, parity, height and history of benign disease
Helzlsouer *et al*.^12^	USA	1974–1991	Cohort	NA	21/42	NA	Pre 1.1 [0.3, 4.1]	Age, time blood drawn fasting
Manjer *et al*.^13^	Sweden	1991–1996	Cohort	41–68	173/438	Post I (<3.78) II (3.78–5.17) III (5.17–7.06) IV (>7.06)	Post 1.34 [0.83, 2.17]	Age, storage time, BMI parity, oophorectomy
Ho *et al*.^14^	Malaysia	2005–2007	Case–control	NA	105/102	Pre I (≤10.595) II (10.595–16.565) III (16.565–30.18) IV (>30.18) Post I (≤6.65) II (6.65–9.66) III (9.66–16.69) IV (>16.69)	Pre 0.45 [0.17, 3.35] Post 0.48 [0.17, 1.36]	Age, time blood drawn, fasting
Tworoger *et al*.^15^	USA	1990–2010	Cohort	25–55	3421/5360	I (≤8.1) II (8.1–11.0) III (11.0–15.7) IV (>15.7)	1.13 [0.99, 1.29]	Age, date and time of day of blood draw, fasting, menopausal status, PMH use, BMI, age atmenarche, history of benign breast disease, family history, age atmenopause
Tikk *et al*.^16^	Europe	1992–2000	Cohort	NA	2250/2250	NA	0.97 [0.54, 1.77]	BMI, full–term Pregnancies, smoking status
Tikk *et al*.^17^	Europe	1992–2000	Cohort	NA	307/307	NA	1.35 [1.04, 1.76]	BMI, smoking status full-term pregnancies

Pre-Premenopausal; Post-Postmenopausal; NA-no report; BMI-Body mass index; NA-not report.

**Table 2:** The results of subgroup-analyses of the relationship between plasma prolaction levels and breast cancer risk according to various factors.

Group	No. Of study	RR (95% CI)	*P* for heterogeneity	I^2^
All	7	1.16 [1.04,1.29]	0.372	7.4%
**Menopause status**				
Premenopause	7	0.99 [0.84,1.16]	0.447	0.0%
Postmenopause	7	1.27 [1.13,1.43]	0.282	19.4%
**ER/PR status**				
ER^+^/PR^+^	3	1.26 [1.04,1.53]	0.007	80.1%
ER^+^/PR^−^	1	1.61 [0.98,2.65]	NA	NA
ER^−^/PR^−^	3	1.00 [0.70,1.43]	0.795	0.0%
**Morphology**				
*In situ*	2	1.29 [1.04,1.61]	0.540	0.0%
Invasive	2	1.32 [1.07,1.63]	0.276	15.9%
**Study design**				
Cohort	6	1.36 [1.18,1.56]	0.832	0.0%
Case-control	1	0.47 [0.20,1.10]	NA	NA
